# The role of hyperparasitism in microbial pathogen ecology and evolution

**DOI:** 10.1038/ismej.2015.247

**Published:** 2016-01-19

**Authors:** Steven R Parratt, Anna-Liisa Laine

**Affiliations:** 1 Department of Biosciences, Metapopulation Research Centre, University of Helsinki, Helsinki, Finland

## Abstract

Many micro-organisms employ a parasitic lifestyle and, through their antagonistic interactions with host populations, have major impacts on human, agricultural and natural ecosystems. Most pathogens are likely to host parasites of their own, that is, hyperparasites, but how nested chains of parasites impact on disease dynamics is grossly neglected in the ecological and evolutionary literature. In this minireview we argue that the diversity and dynamics of micro-hyperparasites are an important component of natural host–pathogen systems. We use the current literature from a handful of key systems to show that observed patterns of pathogen virulence and disease dynamics may well be influenced by hyperparasites. Exploring these factors will shed light on many aspects of microbial ecology and disease biology, including resistance–virulence evolution, apparent competition, epidemiology and ecosystem stability. Considering the importance of hyperparasites in natural populations will have applied consequences for the field of biological control and therapeutic science, where hyperparastism is employed as a control mechanism but not necessarily ecologically understood.

## Introduction

Hyperparasitism, where parasites are themselves infected with parasites, is likely to be a very common phenomenon in nature. However, the impacts of hyperparasitism on the ecology and evolution of microbial pathogens in nature and its cascading effects throughout foodwebs is chronically under-researched. Indeed, we lack a working conceptual framework with which to study the importance of hyperparasites in natural populations. Hypeparasitism may occur obligately—when organisms have specialized to infect other parasites—or facultatively—when generalist or opportunistic parasites infect a number of hosts, some of which are also parasitic. Parasitic microbes are the most probable source of hyperparasites as their small size allows them to use both microbial (Friedman and Crosson, 2012) and multicellular (Hillman and Suzuki, 2004) parasites as hosts. Indeed, entire host–parasite–hyperparasite chains may consist only of microbes (for example see Varon and Levisohn, 1972).

Within an ecological view of parasitism, hyperparasites are superficially analogous to predators, where the intermediate pathogen acts as a herbivore and base-hosts replace primary producers. Thus, as predators are able to shape ecosystem stability through top-down cascades (Hairston *et al.*, 1960), so too hyperparasites may govern pathogen population size and, in turn, host population stability (Gleason *et al.*, 2014). Indeed, the potential for hyperparasite-driven top-down cascades is the basis for their use in both agriculture and medicine as control mechanisms for infectious diseases (Swinton and Gilligan, 1999; Milgroom and Cortesi, 2004; Kiss *et al.*, 2004; Nobrega *et al.*, 2015). However, as argued by Holt and Hochberg, 1998, the tri-trophic relationships in nested parasite chains are fundamentally different to those in predator–prey relationships. First, the population biology and evolutionary potential of parasites differ from predators because they are smaller, often more numerous than their host, have shorter generation time than their host, and are often dependent upon a host individual rather than predominantly free-living. Second, hosts can bidirectionally change class between being infected and uninfected by either acquiring or clearing infection, whereas predation is typically irreversible. Therefore, the role of hyperparasites within ecosystems will not be adequately captured with existing tri-trophic models.

Some theoretical models do explicitly address both general and system-specific hyperparasite effects on pathogen and host populations (Beddington and Hammond, 1977; van der Kamp and Blenis, 1996; Holt and Hochberg, 1998; Taylor *et al.*, 1998; Taylor, 2002; Morozova *et al.*, 2007). However, empirical studies of the prevalence and effects of micro-hyperparasites in many natural systems remain relatively rare. This scarcity of data is most likely owing to the inherent crypsis of micro-hyperparasites, which are small and often intracellular in both micro- and macro-hosts. However, the availability of genetic resources and reliable molecular screening techniques make it increasingly feasible to study the prevalence, diversity and impacts of micro-hyperparasites in natural host–pathogen systems. For example, deep sequencing technologies and metagenomic approaches can reveal the diversity of micro-organism communities carried by hosts (for example see Chandler *et al.*, 2015) and describe the diversity of nested chains of microbiota (see De Paepe *et al.*, 2014). Experimental approaches can then elucidate the antagonistic, commensal or mutualistic nature of such relationships and their possible cascading impacts.

In this review we examine the existing evidence that hyperparasitism has a critical role in the evolution and epidemiology of microbial pathogens. We conclude that these ecological and evolutionary effects may combine to shape patterns of microbe and host diversity and affect natural disease dynamics. We call for more research on the impacts of micro-hyperparasites in natural systems, and for a conceptual framework for nested parasite chains and their consequences to be developed.

## Hyperparasites and pathogen virulence

Theory predicts that pathogens evolve virulence levels towards an optimal evolutionary stable strategy (ESS) through a trade-off between transmission and host-damage (Ewald, 1983; Frank, 1992; Alizon *et al.*, 2009; Doumayrou *et al.*, 2013). This may be mediated by numerous factors, such as mixed routes of transmission (Ewald, 1987; Turner *et al.*, 1998; Stewart *et al.*, 2005), host life-history (Gandon *et al.*, 2001) and coinfection (Alizon and van Baalen, 2008), or distorted in systems not yet at equilibrium (Day and Proulx, 2004; André and Hochberg, 2005). However, hyperparasitism has the potential to perturb realized virulence levels away from the ESS, therefore affecting observable pathogen virulence in nature and potentially acting as a selective pressure on pathogen virulence evolution.

Hyperparasite infection can attenuate a pathogen's realized virulence below the ESS if they directly debilitate its growth or ability to produce and deploy virulence factors; a phenomenon broadly termed hypovirulence. Many viral infections of plant pathogenic fungi have been shown to induce hypovirulence (Nuss, 2005), arguably the best researched of which is CHV1 virus in the causative agent of chestnut blight, *Cryphonectria parasitica*. Here, the hyperparasite reduces pathogen growth, which subsequently curtails damage to the tree host and significantly reduces the pathogen's devastating effect on chestnut populations (Choi and Nuss, 1992). A similar effect is exhibited by filamentous phage ϕRSM infecting the bacterial wilt pathogen, *Ralstonia solanacearum.* Here, phage infection reduces the bacteria's virulence in host plant tissues by limiting the expression of bacterial virulence factors (Addy *et al.*, 2012). In these cases the hyperparasite actively reduces the damage caused by the pathogen, thus reducing the pathogen's virulence below the evolved optima. It is the ability of micro-hyperparasites to reduce realized pathogen virulence in this way that has led them to be deployed as biocontrol measures in agriculture and as phage-therapy for treating bacterial infections in humans (Nobrega *et al.*, 2015). However, we have very limited empirical evidence for the impacts of hyperparasite-induced hypovirulence on pathogen virulence evolution. For example, does pressure from hypovirulence-inducing hyperparasites select for increased virulence evolution in pathogens by way of compensation? If this occurs, hyperparasites may actually select for higher virulence in pathogens by directly attenuating the damage a pathogen inflicts on its host. This may create a problem when assessing observable virulence in natural systems in which hyperparasites are present. Do we see patterns of virulence that are the product of direct action by unseen hyperparasites, compensatory evolution on the part of the host, or a combination of the two?

When low virulence is optimal, infection by a hypovirulence-inducing hyperparasite may be beneficial for pathogens as it reduces realized virulence levels closer to the ESS. This may allow a virulent pathogen to invade host populations from which it would otherwise be excluded. Such a phenomenon may be a precursor to the evolution of mutualistic symbioses between microbes. For example, the heritable insect symbiont *Hamiltonella defena* loses its defensive properties and becomes more virulent when purged of APSE prophage, destabilizing the microbe's interaction with its arthropod host (Weldon *et al.*, 2013). This, and similar, microbe–microbe associations potentially evolved from an ancestral hypovirulence-inducing hyperparasitism.

There is also strong evidence that micro-hyperparasite infection, in addition to inducing hypovirulence, can directly increase pathogen virulence (that is, induce hypervirulence). To date, one of the best described examples of hypervirulence comes from phage-encoded virulence factors in bacterial pathogens (Brussow *et al.*, 2004). For example, λ and CTXφ prophage encode STX and CTX toxins in *Escherichia coli* and *Vibrio cholera*, respectively, and similar toxin-encoding prophages have been described in other pathogenic bacteria including *Staphylococcus aureus*, *Salmonella entericia* and *Streptococcus pyogene* (Fortier and Sekulovic, 2013). Furthermore, several virulence factors have been identified on plasmids in pathogenic bacteria such as *E. coli* (Johnson and Nolan, 2009). In situations in which high virulence is adaptive these mobile elements may act as mutualists (see ‘biological weapon hypothesis' in Dheilly *et al.*, 2015). However, their relationship with the pathogen host will be antagonistic if high virulence is maladaptive or, as in the case of λ and CTXφ prophage, toxin expression is linked with pathogen cell death (Abedon and LeJeune, 2005). Hyperparasite infection may also cause hypervirulence by eliciting stress responses. External stressors can alter pathogen life-history traits in favor of reproduction (for example see Carter *et al.*, 2013). If reproduction and virulence are positively linked, as proposed by the transmission-virulence trade-off theory (Ewald, 1987; Frank, 1996; Alizon *et al.*, 2009), this may cause pathogens to inflict greater damage on their hosts due to hyperparasite-induced stress.

Where hyperparasites shift pathogen virulence away from the ESS we expect resistance evolution to occur. Indeed, there is evidence that pathogen strains can vary in their ability to resist hyperparasite attack in natural populations (Bryner and Rigling, 2011, Parratt and Laine *in prep*). The evolution of resistance may impact upon the evolution of virulence if the two traits are genetically correlated. For example, pathogenic bacteria selected to resist phage attack have repeatedly shown a loss of virulence towards their hosts (Laanto *et al.*, 2012; Friman *et al.*, 2013; Hosseinidoust *et al.*, 2013; Le *et al.*, 2014). In addition, models of co-evolution in similar three-species interactions suggest that a genetic correlation between interaction traits such as resistance and virulence levels can lead to cyclical maladaptation in the intermediate organism and destabilize tri-partite interactions (Nuismer and Doebeli, 2004). Thus, hyperparasitism may impose additional selective pressures on pathogens, which perturb other co-evolutionary interactions and may shape natural patterns of disease severity.

The complexities of studying virulence evolution and observable virulence levels in nested parasite chains have many parallels in the field of coinfection dynamics. Similar to the study of coinfections, one of the major challenges in understanding the effects of hyperparsites will be to distinguish whether hyperparasites drive virulence evolution (that is, whether hyperparasitism selects for more or less virulent pathogen strains) or affect the expression of virulence (whether infection outcome is more or less virulent in the presence of a hyperparasite (Alizon, 2013)). Although hyperparasitism fundamentally differs from coinfection, for example, hyperparasites are unlikely to directly interact with the base-host, the fields are likely to share common conceptual ground.

## Hyperparasite adaptations to pathogens

**Figure 1:**
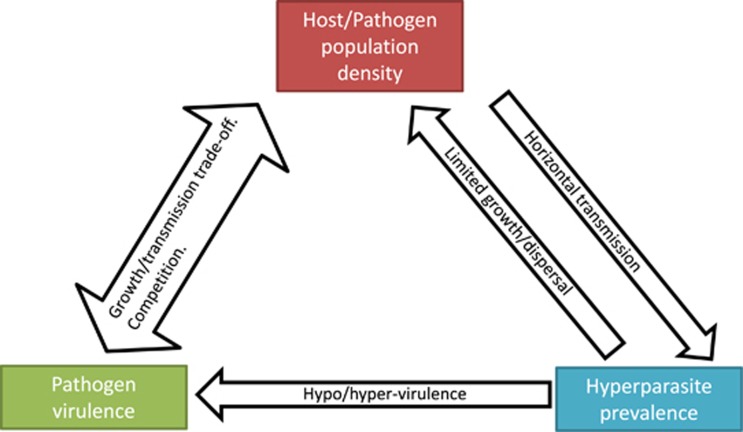
Potential interactions between pathogen density, virulence and hyperparasite prevalence. Hypovirulence may disrupt the adaptive benefit of pathogen virulence strategies under varying levels of density. This in turn can have knock-on effects for the hyperparasite itself by limiting pathogen population size and thus the opportunity for horizontal transmission. Some of this conflict can be ameliorated if hyperparasites utilize vertical transmission (Taylor, 2002). Arrows indicate the direction of effect.

An enduring concept from the field of virulence evolution is that higher virulence is likely to evolve in dense host populations, where opportunities for horizontal transmission are common (Fine, 1975; Ewald, 1987). In his models of pathogen–hyperparasite systems, Taylor *et al.* (1998; 2002) recognized that such a coupling of increased virulence with horizontal transmission may result in conflict for hyperparasites. Hypovirulence-inducing hyperparasites can easily spread among individuals in a dense pathogen population, but will inherently reduce pathogen virulence and therefore horizontal transmission rate as they do so. Therefore, hypovirulence induction may ultimately limit hyperparasite transmission by reducing pathogen population density. This would curtail the invasion potential of a hypovirulence-inducing organism, and thus any top-down cascade effect may be inherently self-limiting. The models by Taylor (2002) were built for the Chestnut blight-CHV1 system, where both vertical and horizontal transmission of the hyperparasite is possible. He argues that such a mixed mode of transmission may alleviate similar conflicts (Taylor, 2002). However, the extent to which other hyperparasites exhibit mixed-mode transmission strategies in nature is poorly explored (Ebert, 2013). Similarly, hypervirulence-inducing hyperparasites may also be self-limiting. Maladaptively high virulence may result in increased host death and thus a reduction in pathogen density. This would, in turn, make transmission more difficult for the hyperparasite and so again curtails their spread. Whether there exists stable equilibrium between pathogen virulence, host density and hyperparasite transmission and their effects in nature is almost unexplored. Yet the interconnections of these factors may be key determinates of observed epidemics of infectious disease (Figure 1).

Co-evolution in natural hyperparasite systems is likely to be highly complex. The few existing models of hyperparasite-pathogen dynamics tend to predict variable outcomes depending upon the initial parameter values (Holt and Hochberg, 1998; Taylor *et al.*, 1998; Morozova *et al.*, 2007). The evolutionary outcome of host–pathogen–hyperparasite interactions will also be dependent upon their species-specificity, which has only been explored in some systems (for example see Liang *et al.*, 2007; Pintye *et al.*, 2012). For example, a generalist hyperparasite may ameliorate the effects of the conflicts detailed above by utilizing several host species and so being host-density independent. Furthermore, pathogen–hyperparasite co-evolution does not occur in isolation, but also in tandem within an evolutionarily active host background. However, little attention has been given to any interactions between hyperparasitism and base-host resistance, and whether such relationships are mutualistic or antagonistic (Box 1).

There is evidence that pathogens and hyperparasites are engaged in co-evolutionary relationships, as infection success can vary between genotype combinations (Bryner and Rigling, 2011, Parratt and Laine *in press*). Furthermore, there is evidence that hyperparasite infection can alter the genetic structure of pathogen populations (Springer *et al.*, 2013) and the genomics of virulence (Johnson and Nolan, 2009), indicating potential for reciprocal molecular evolution and local adaptation. Further research should focus on testing for signals of co-evolution in natural host–pathogen–hyperparasite systems and its consequences for realized virulence and disease epidemiology.

## Hyperparasites and coinfections

Above we have discussed how pathogen populations at high density are likely to favor the horizontal transmission of hyperparasites. However, dense host populations are also likely to produce coinfections of mixed pathogen strains in single-host individuals. This duality is non-trivial, as the action of hyperparasites may also mediate the outcome of competition between coinfecting pathogen strains.

Coinfections are thought to be common in nature (García-Arenal *et al.*, 2001; Lopez-Villavicencio *et al.*, 2007; Karvonen *et al.*, 2012). Theory predicts that competition between coinfecting pathogen genotypes may lead to the expression and evolution of virulence levels that would be sub-optimal in single-infection scenarios (May and Nowak, 1995; Alizon and van Baalen, 2008). This has been demonstrated in several systems, where mortality is higher (Milbrath *et al.*, 2015) and within-host disease burden and between-host transmission increases (Susi *et al.*, 2015) under coinfection. However, the apparent competition between coinfecting pathogen genotypes can be generated by external factors, and is not necessarily a product of direct pathogen–pathogen interaction. For example, host immunity may filter pathogen genotypes and thus alter the outcome of coinfection (Råberg *et al.*, 2006; Cobey and Lipsitch, 2013). Indeed, host immunity can inadvertently select for increased virulence if avirulent pathogen strains are also more susceptible than virulent strains to immune effectors (Råberg *et al.*, 2006). Furthermore, parasites themselves have been implicated in driving apparent competition when resistance varies among host species. Resistant hosts can act as a reservoir of pathogens that debilitate more susceptible individuals (Cobb *et al.*, 2010). In a similar way, micro-hyperparasites may produce apparent competition between pathogen strains. If virulence toward the host and resistance against the hyperparasite are correlated, but the shape of this correlation varies between pathogen strains, then virulent pathogen genotypes may be more or less affected when hyperparasites attack. This would result in apparent competition between pathogen genotypes driven by hyperparasite-induced filtering. There is some evidence that pathogen strains vary in their levels of susceptibility to hyperparasite infection (Bryner and Rigling, 2011, Parratt & Laine *in prep*), but this has not been explored in the context of coinfection and apparent competition. Research should now explore how pathogens with variable levels of resistance to hyperparasites perform when in competition, and whether the outcome of coinfections can be moderated by hyperparasites. If hyperparasites can alter the outcome of coinfections then they will impact on pathogen population structure and drive the spread of particular pathogen genotypes.

## Hyperparasites and disease epidemiology

The outcome of co-evolutionary relationships between micro-hyperparasites and pathogens may significantly affect the dynamics of infectious disease. Theory predicts that the presence of hyperparasite-infected pathogen strains can affect the stability of trophic systems (Holt and Hochberg, 1998) and alter the conditions under which pathogens may successfully invade (Taylor *et al.*, 1998). However, evidence of these effects is confined to a few natural systems (see Table 1 for examples).

When hyperparasites induce hypovirulence they can limit both the severity and transmission of infectious disease. This effect is best exemplified by the mycovirus CHV1 in populations of the chestnut blight fungus *C. parasitica*. When efficiently transmitted through pathogen populations, CHV1 allows chestnut trees to survive the introduction of *C. parasitica,* an effect which has manifested strongly in Europe and to a lesser degree in North America (Milgroom and Cortesi, 2004). *C. parasitica* invasions in North America have driven chestnuts from being the dominant canopy trees to undergrowth community members, an effect which is partially reversed in CHV1-affected populations (Davelos and Jarosz, 2004). Where the virus has allowed tree populations to recover, it has also fundamentally changed the genetic structure of the pathogen population (Springer *et al.*, 2013) and so has impacted upon both host and pathogen populations. Other systems have shown similar hyperparasite-induced control of disease. *Ampelomyces quisqualis,* is an obligate mycoparasite that infects a diverse array of powdery mildew pathogens of agriculturally important and naturally occurring plants (Liang *et al.*, 2007; Angeli *et al.*, 2012; Pintye *et al.*, 2012; Tollenaere *et al.*, 2014). *A. quisqualis* can reduce the dispersal and virulence of mildews during summer growing seasons and has been positively associated with pathogen extinction during overwintering, thus potentially fundementally altering the pathogen population dynamics (Verhaar *et al.*, 1996; Kiss, 2008; Angeli and Puopolo, *et al.*, 2012; Tollenaere *et al.*, 2014). Similarly, hyperparasitic Sputnik Virophage infection has been shown to regulate the population dynamics of phycodnaviruses in their algal hosts in Antarctic lakes, ultimately reducing base-host mortality (Yau *et al.*, 2011). Furthermore, JSF4 phage can limit epidemics of *Vibrio cholera* in human populations (Faruque *et al.*, 2005). Population-level effects of hyperparasites can also occur in *Ophiocordyceps*-infected ants; *Ophiocordyceps spp.* manipulate infected hosts to seek out vantage points where they die and thus the fruiting bodies of the fungus have optimal wind dispersal to nearby colonies (Pontoppidan *et al.*, 2009). Fungal infection is extremely virulent at the individual level because host death is required for transmission. However, evidence suggests that long-term infections at the ant colony level are facilitated through infection of the immature *Ophiocordyceps* by castrating hyperparasitic fungi. A synthesis of life-stage observations and disease dynamic modeling in this system suggests that the hyperparasite reduces the number of successful infections at an individual scale, but in doing so facilitates disease persistence at the colony level (Andersen *et al.*, 2012).

This handful of examples likely represent a broader array of micro-hyperparasites and their effects on pathogenic organisms in nature. Given the variety of potential co-evolutionary outcomes presented above, the potential individual- and population-level impacts of hyperparasite infection on pathogens will be diverse. Unraveling these effects may offer unique insight to an unexplored facet of natural disease dynamics.

## Conclusions

Here we have highlighted the limited evidence that micro-hyperparasites can fundamentally affect the outcome of disease. These effects can manifest at the individual host level, alter the outcome of coinfecting pathogen strains, and ultimately mediate the population dynamics of infectious diseases. Although some early models have approached these dynamics from both an ecological and evolutionary standpoint, we still lack a universal framework for hyperparasite-driven disease ecology. Furthermore, our empirical exploration of the dynamical effects of hyperparasites in natural systems is limited to only a few organisms. Given that the majority of parasites and, by extension, hyperparasites are likely to be micro-organisms, it is likely that this oversight has been because of the cryptic role these organisms have had. Now, with a plethora of molecular resources available, researchers should fully examine the diversity and effects of microbial hyperparasites in nature.

## Figures and Tables

**Table 1:** Examples of key microbial hyperparasites and their effect on pathogens

*Hyperparasite*	*Pathogen(s)*	*Hosts*	*Key effects*	*References*
Cryptophonectria hypovirus-1 (CHV1)	*Cryphonectria parasitica*	Chestnut trees (genus *Castanea*)	Reduces pathogen growth rate and virulence to host. Alters genetic structure of *C. parasitica* populations. Allows tree host populations to recover to near disease-free demography	Hillman and Suzuki (2004); Milgroom and Cortesi (2004) Springer *et al.* (2013) Davelos and Jarosz (2004)
*Ampelomyces quisqalis*	*Podosphaera spp.* *Erysiphe spp.* *Oidium spp.* *Arthrocladiella mougeotii* *Golovinomyces spp.* *Sphaerotheca fuliginea*	Numerous plant species including: *Plantago spp.* Cucumber Grape Apple Strawberry	Reduced pathogen growth Reduced pathogen overwintering success Reduced pathogen sporulation Rescues host plant chloroplast from deterioration	Verhaar *et al.* (1996) Tollenaere *et al.* (2014) Falk *et al.* (1995) Shishkoff and McGrath (2002) Angeli, *et al.* (2012) Abo-Foul *et al.* (1996) Romero *et al.* (2003)
Unknown fungal hyperparsites	*Orphycordyceps camponoti-rufipedis*	Ant: *Camponotus rufipes*	Castrates immature fruiting body and reduces viability of spores. Limits transmission effeciency of the pathogen.	Andersen *et al.* (2012)
APSE phage	*Hamiltonella defensa*	Aphid: *Acyrthosiphon pisum*	Reduces bacterial abundance in aphid host. Phage loss associated with fitness reduction in *H. defensa* infected aphids. Phage presence linked to protective property of *H. defensa* against aphid's natural enemies.	Weldon *et al.* (2013)
JSF4 bacteriophage	*Vibrio cholera*	Human	Phage lysis associated with self-limiting bacterial epidemic.	Faruque *et al.* (2005)
LESϕ prophage	*Pseudomonas aeruginosa*	Human	Phage lysis associated with bacterial population size regulation.	James *et al.* (2015)

## Box 1. Host resistance and hyperparasite attack: potential for mutualism or antagonism

Where evolved pathogen virulence is high there exists the potential for co-evolutionary relationships between hypovirulence-inducing hyperparasites and base-hosts. Whether these relationships become mutualistic or antagonistic may depend on the unique conditions of each system, the prevalence of hyperparasites and their specificity.

Mutualisms:

Mutualism between host and hyperparasite may occur through two mechanisms:

1. When hyperparasite infections are common they may reduce the strength of selection on host resistance by dampening the selective pressure of pathogen virulence.
2. Hyperparasite infection and host immunity work synergistically to reduce pathogen virulence. This is the case in the chestnut blight system, where CHV1 induces growth retardation of the fungus that then allows the immune response of the trees to isolate and encapsulate invasive hyphae before they do terminal damage (Davelos and Jarosz, 2004).

In these scenarios hyperparasite effects may distort observed patterns of host–pathogen co-evolution in nature. If host and pathogen interactions are examined in isolation from the hyperparasite then hot-spots of antagonistic selection will be masked.

Antagonism:

In ecological time host resistance reduces parasite transmission and therefore population size. This is detrimental to the hyperparasite as it limits the resources they exploit (pathogen population size) and the potential for horizontal transmission. Thus, further reduction in pathogen virulence caused by hypovirulence will be maladaptive to both the pathogen and hyperparasite and so host resistance and hyperparasite hypovirulence may be under opposing selection regimes. Where host resistance is efficient then hyperparasites should be selected to minimize hypovirulence effects so as not to further debilitate the pathogen population on which they rely.
